# Comparative Analysis of NiTi Instruments with Different Alloy Treatments

**DOI:** 10.3390/ma17194817

**Published:** 2024-09-30

**Authors:** José Aranguren, Felipe Oliveros-Porras, Ana Ramírez-Muñoz, Irene Pérez, Marcela Salamanca-Ramos, Karim Aazzouzi-Raiss, Alejandro R. Pérez

**Affiliations:** Department of Endodontics, Rey Juan Carlos University, 28933 Madrid, Spain; josearanguren@hotmail.com (J.A.); felipe_7523_oliveros@hotmail.com (F.O.-P.); anaramimu96@gmail.com (A.R.-M.); ipereztejeiro@gmail.com (I.P.); marce0415@gmail.com (M.S.-R.); aar_karim@hotmail.com (K.A.-R.)

**Keywords:** nickel–titanium instruments, root canal instrumentation, cyclic fatigue resistance, endodontic file systems

## Abstract

This study aims to compare the cyclic fatigue resistance of nickel–titanium (NiTi) endodontic instruments, focusing on the impact of various alloy treatments and manufacturing processes across different generations of these instruments; Twenty instrumentation systems from different generations, comprising both continuous and reciprocating motion designs, were tested. Four hundred instruments underwent cyclic fatigue testing using an INSTRON machine, with the time and number of cycles to fracture (NCF) recorded for each instrument. Statistical analyses were performed to compare the fatigue resistance between systems, generations, and motion types; Instruments treated with advanced thermal processing, such as Excalibur, Reciproc Blue, and TruNatomy, demonstrated superior resistance to fracture, whereas systems like Protaper Universal, K3XF, and 2Shape showed the lowest resistance. Reciprocating instruments generally exhibited higher cyclic fatigue resistance than continuously rotating instruments; Technological advancements in NiTi instrument design, especially the implementation of heat-treated alloys, have improved cyclic fatigue resistance, enhancing the safety and efficiency of endodontic treatments. Reciprocating systems, in particular, exhibit superior fracture resistance, suggesting their greater utility in challenging clinical conditions.

## 1. Introduction

The near equiatomic nickel–titanium (Nitinol/NiTi) alloys were first developed by Buehler and Wiley of the US Naval Ordnance Laboratory in the 1960s, and this great combination was first used in endodontics in late 1980 [1], revolutionizing root canal instrumentation by addressing limitations of stainless steel files, such as zipping and ledges [2,3]. Early innovations, like the ProFile series (Dentsply, Charlotte, Pennsylvania, USA), formed the first generation of NiTi rotary instruments, allowing for continuous rotary motion in curved canals. As the second generation emerged in the late 1990s, instruments like ProTaper Universal (Dentsply Maillefer) improved efficiency with active cutting edges and fewer files required [4].

Advancements in NiTi metallurgy marked the third generation, with heat-treatment processes enhancing fatigue resistance. Instruments made from heat-treated M-wire, such as Vortex Blue (Dentsply Maillefer), demonstrated greater durability and flexibility [5,6,7,8]. The fourth generation introduced single-file reciprocating systems, like WaveOne (Dentsply-Sirona, Baillagues, Switzerland) and Reciproc (VDW, Munich, Germany), further improving safety and performance [9,10,11,12].

Fifth-generation instruments, such as TRUshape (Dentsply Tulsa Dental Specialties, Tulsa, OK, USA) and EDGEendo (Edge Endo, Albuquerque, NM, USA), focused on preserving pericervical dentin with smaller tapers [13,14,15], while the latest, sixth-generation instruments, like BlueShaper (Zarc4endo, Gijón, Asturias, Spain) and Protaper Ultimate (Dentsply-Sirona, Baillagues, Switzerland), combine multiple alloys to enhance both fracture resistance and cutting efficiency [16]. Despite significant progress, challenges remain regarding instrument fracture in complex root canal anatomies.

**Figure 1:**
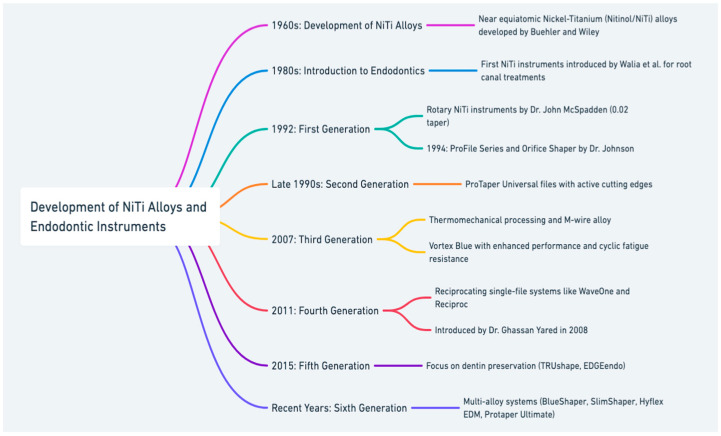
This diagram presents the historical development of nickel–titanium endodontic instruments, from their inception in the 1960s to recent innovations.

To better understand the evolution of NiTi instruments, refer to the attached graphic illustrating their development over time (Figure 1). This study compares twenty NiTi systems across various generations, evaluating their resistance to cyclic fatigue and time to fracture.

## 2. Materials and Methods

### 2.1. Endodontic Instrumentation Systems

As no human or animal teeth, tissues, or cells were used, obtaining an ethics committee approval for the conduct of the present study was unnecessary. Second-, third-, fourth-, fifth-, and sixth-generation continuous and reciprocating endodontic instruments were evaluated. These instruments were selected based on their prevalence in the literature and relevance to the research question. The physical characteristics of the individual systems are outlined in the Supplementary Table S1.

### 2.2. Design of Files and Cyclic Fatigue

**Figure 2:**
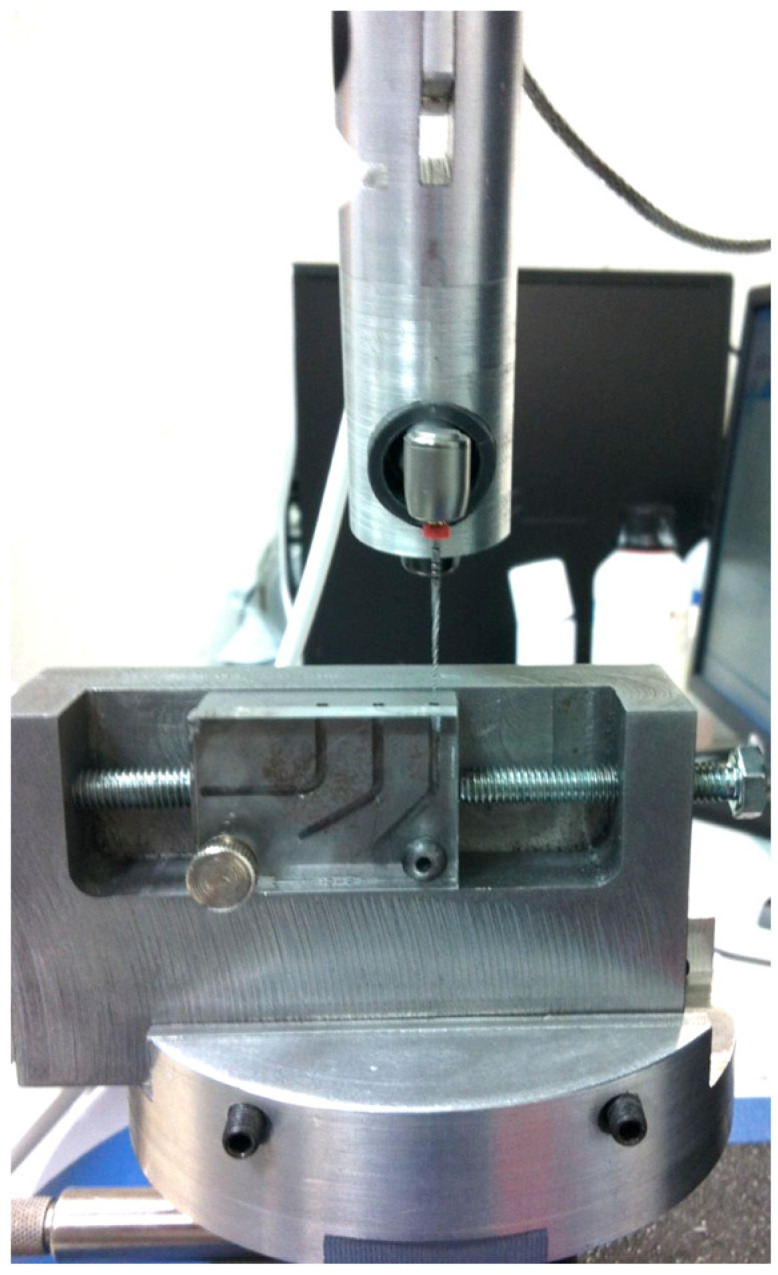
Photo of the device used to perform cyclic fatigue tests with the different instrumentation systems.

A total of 400 instruments were included in the study, with 20 files for each instrumentation system. The instruments underwent a cyclic fatigue test using an INSTRON 3345 power machine (Instron, Norwood, MA, USA) and Bluehill Lite computer software (v.2.18, 2005) (Figure 2). Each instrument was inserted into an artificial stainless steel canal with the following dimensions: 1.5 mm width, 26 mm length, 60° curvature, and 5 mm radius. To minimize friction, a lubricating spray was applied between experiments [17,18].

The experimental setup involved positioning the instruments at the canal entrance, inserting them 22.5 mm to avoid contact with the metal device stop, and activating them using a cordless endodontic motor (E-connect, Eighteeth, Shenzhen, China). The motor was secured in a stainless steel fixture within the testing machine, with instruments aligned at the canal entrance.

All instruments were loaded at the manufacturer’s recommended rotational speed and torque. The time to fracture for each instrument was calculated in seconds using Bluehill Universal v4.37 software graphs, displaying load force values and time from instrument activation to fracture. The number of cycles to fracture (NCF) was calculated using the formula: NCF = (rpm × time to fracture)/60.

**Figure 3:**
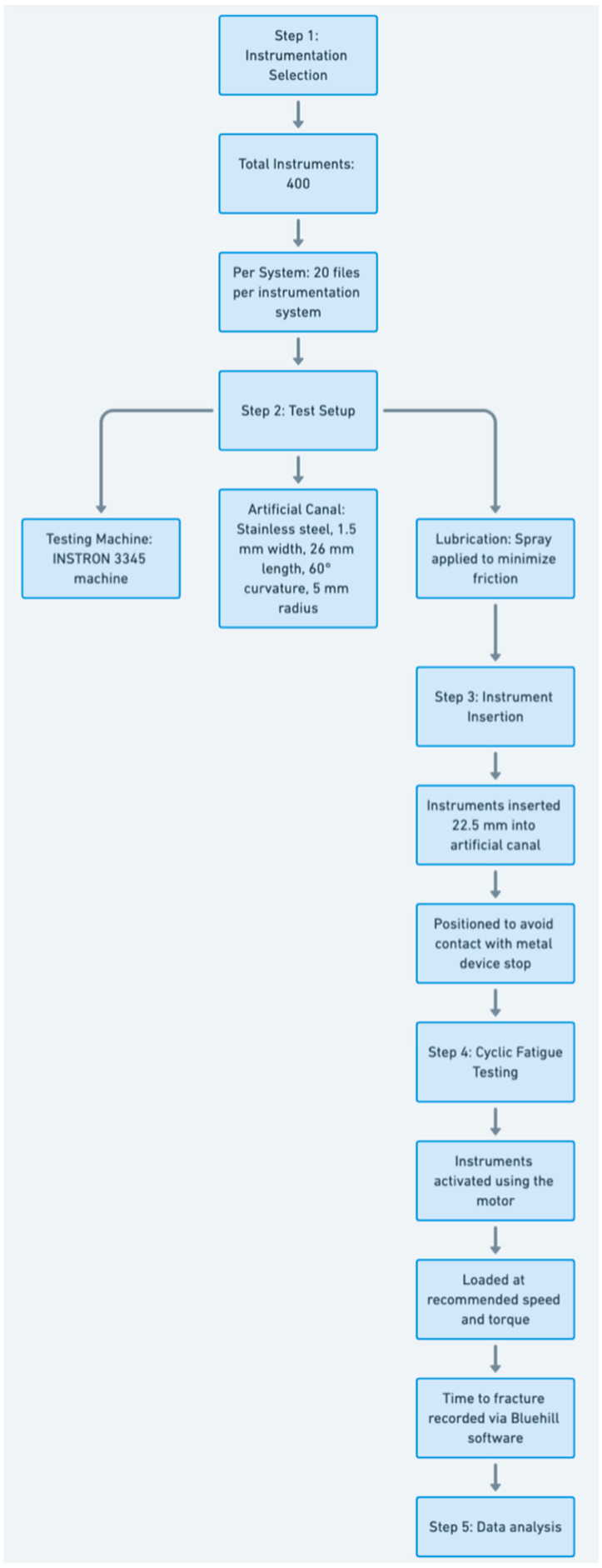
Diagram showing the stages the cyclic fatigue tests.

Following cyclic fatigue tests, the data collected from separated instruments were entered into an Excel spreadsheet (Microsoft Corporation, Redmond, WA, USA) for further comparison. Figure 3 shows the stages performed during the cyclic fatigue tests.

### 2.3. Statistical Analysis

The Shapiro–Wilk test was employed to assess the normality of the obtained data. Since the results indicated a normal distribution, the ANOVA test was performed to compare the time to fracture and NCF between the different instruments. This comparison was conducted with instruments of the same generation and between different generations. Furthermore, a comparison between reciprocal systems was also performed. Finally, a comparison was made between systems with continuous rotation and systems with reciprocal motion. The statistical program SPSS (Statistical Package for the Social Sciences 21.0; IBM Brasil, SP, Brazil) with a significance level of 5% was used for all analyses.

## 3. Results

The instrument characteristics are comprehensively presented in the Supplementary Table S1. Table 1, Table 2 and Table 3 show the results regarding NCF and time to fracture for each generation, respectively. 

**Figure 4:**
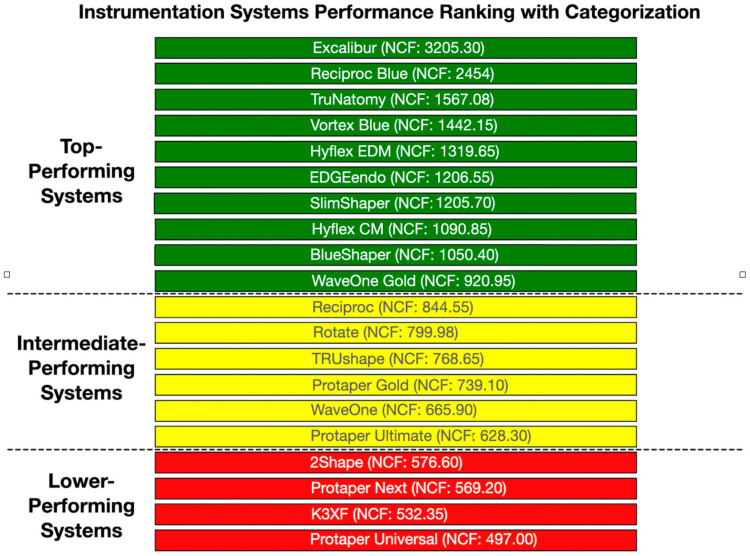
Sequence of performance classification of instrumentation systems based on their NCF (Number of Cycles to Fracture) values. The systems are categorized into three groups: Top-Performing (Green), Intermediate-Performing (Yellow), and Lower-Performing (Red). Each data point represents a system labeled with its name and corresponding NCF value, enabling direct comparison of its performance.

**Figure 5:**
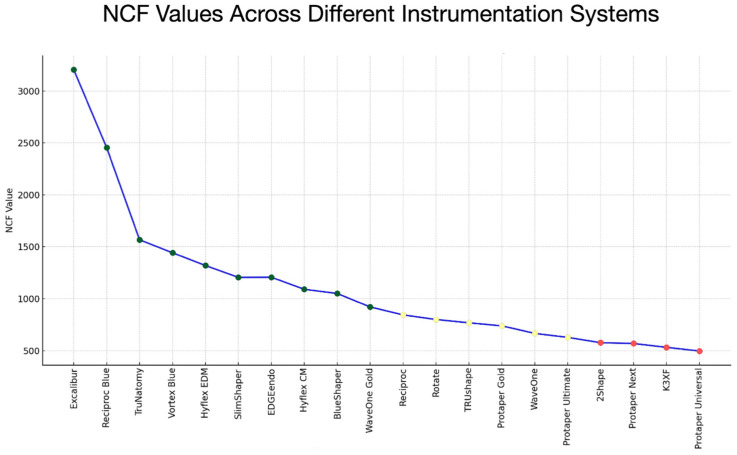
Chart displaying the values of NCF for each instrumentation system, arranged according to their performance categories: Top-Performing (Green), Intermediate-Performing (Yellow), and Lower-Performing (Red). Each system is represented by a point on the line, and the colors of the points indicate their performance category, providing a clear visualization of how each system’s NCF compares to others.

Figure 4 and Figure 5 provide a detailed description of the comparisons between the different instrumentation systems regarding their NCF fracture resistance, from those exhibiting the highest fracture resistance to those with intermediate fracture resistance and concluding with those showing the poorest fracture resistance.

## 4. Discussion

One of the main goals of root canal treatment is to prepare as many areas as possible within the root canal system, as those areas left uninstrumented will harbor necrotic debris and biofilms, which are particularly critical in the apical third and may cause treatment failure [19]. Fracture of instruments in the root canal makes access to this apical region difficult and hinders proper disinfection and bacterial removal.

To analyze the cyclic fatigue resistance of the selected instruments in this study, an artificial canal made of stainless steel, a material commonly used in studies evaluating fracture behavior [20], was used, allowing standardization and comparison of the results.

The findings of this study reveal that the instruments with the highest cyclic fatigue resistance are Excalibur (NCF: 3205.30) and Reciproc Blue (NCF: 2454.00), followed by the Trunatomy, Vortex Blue, Hyflex EDM, SlimShaper, EDGEendo, Hyflex CM, Blueshaper, and WaveOne Gold systems, which demonstrate higher cyclic fatigue strength than the other instruments. The instruments with the worst performance are 2Shape, Protaper Next, K3XF, and Protaper Universal.

The results show that reciprocating and continuous heat-treated systems yield the best results. Second- and third-generation instruments offer lower fracture resistance. The introduction of heat treatment in fourth-, fifth-, and sixth-generation instruments has improved the resistance to cyclic fatigue in the instruments analyzed in this study. 

Nehme et al. investigated the cyclic fatigue resistance of the same instrument with and without heat treatment. They found that heat treatment increased cyclic fatigue resistance [7]. The same instruments with different alloys, primarily among those with heat treatment, have significantly different levels of fracture resistance. These findings help explain the differences between systems that only differ in their alloy while the rest of their physical characteristics remain the same.

A comparison of the different systems with and without heat treatment shows an increase in NCF for the heat-treated generations. Reciproc and Reciproc Blue show an NCF of 844.55 and 2454.00, respectively. Similar results were obtained by Al-Obaida, et al. [21], who analyzed the resistance of different reciprocal systems and found that Reciproc Blue is more resistant to cyclic fatigue than Reciproc, which has a higher resistance than the Twisted File system.

These results are consistent with the present study, in which Reciproc Blue and Excalibur have the highest resistance, probably because, in addition to heat treatment, the reciprocating movements offer a higher resistance to cyclic fatigue [11,12]. 

An essential feature contributing to the superior performance of the Excalibur relative to other reciprocating systems could be its taper. Whereas other reciprocating systems typically possess greater tapers in the range of 7–8%, the Excalibur system exhibits a lower taper (5%), potentially enhancing its fracture resistance. Additional attributes such as helical angle and cross-sectional configuration may also contribute to improved performance. Nevertheless, further investigations are warranted to systematically assess various characteristics of the Excalibur system in contrast to alternative reciprocating systems, and thereby advance the comprehension of the diverse physical properties inherent in such systems.

Incorporating gold heat treatment into the WaveOne system significantly increases its fracture resistance so that WaveOne Gold is no longer inferior to other systems in terms of cyclic fatigue resistance. Adıgüzel and Capar [22] observed that WaveOne Gold withstood twice as many cycles as WaveOne at 60° curvatures and three times as many cycles at 90° curvatures.

The heat treatment of the Protaper systems also significantly increases fracture resistance, with Protaper Gold having almost twice as many cycles to fracture compared to Protaper Universal. In other studies, the fracture strength of Protaper Gold was found not to vary significantly at different temperatures, in contrast to Protaper Universal, whose cyclic fatigue strength decreases with increasing service temperature [23].

What is remarkable about our results is that Protaper Ultimate has a lower cyclic fatigue resistance than Protaper Gold. As they are instruments with the same alloy and taper, the differences may be attributed to the number of spirals, helical angles, and tip geometry. A recent study [16] showed that Protaper Ultimate instruments have lower torsional strength and greater flexibility than their counterparts; however, further research is required to confirm the results of the current study.

It is important to emphasize that the heat treatment of the systems has changed the perspective of root canal instruments in terms of flexibility and fracture resistance. For example, safe instrumentation is possible even in more complex cases thanks to the heat-treated instruments. In a clinical study [8] with WaveOne Gold, no instrument fractures were observed in the 1104 instruments used in root canal preparation of maxillary and mandibular molars with curvatures of less than 45° when used strictly according to the manufacturer’s recommendations and in a single clinical case. 

The present study found that the Hyflex EDM system delivers results comparable to Vortex Blue, SlimShaper, Trunatomy, and EDGEendo. However, Hyflex CM shows significantly worse outcomes than Hyflex EDM (*p* < 0.05). This difference between the two systems is probably due to the different alloys of both systems. Hyflex EDM has a higher NiTi content in the martensitic phase [24] compared to the CM wire alloy of the Hyflex CM system.

TruNatomy exhibits greater fracture toughness than first-generation instruments such as Protaper Next [25]. The differences may be primarily related to the heat treatment of TruNatomy and the smaller tapers compared to the Protaper Next system.

Although cyclic fatigue tests were performed statically in this study, some studies have analyzed the behavior of different instruments dynamically and statically. Reciprocating instruments were found to have higher resistance, both statically and dynamically; NCF values increased significantly in continuously rotating instruments, whereas resistance did not increase in reciprocating instruments [12]. Therefore, it should be considered that the continuous rotation instruments investigated in this study may have higher NCF values when analyzed dynamically, i.e., in motion.

To our knowledge, this is the first study to assess the NCF and time to fracture of instruments from different generations, including reciprocating and continuous rotation systems (20 systems in total). However, this study has some limitations. First, we examined the behavior of the different instruments at room temperature (20 °C), not at body temperature (37 °C), and they were also not soaked in sodium hypochlorite, which could alter our results.

While some studies found that the cyclic fatigue resistance of Vortex Blue [26] and Endosequence [27] instruments decreased with increasing temperature, others, such as Plotino et al. [23], observed that Protaper Gold showed no differences in cyclic fatigue resistance when the temperature increased from 20 °C to 35 °C. Similarly, Keles et al. [28] observed that the NCF of Reciproc Blue 25/.08 was not affected by temperature changes or using hypochlorite instead of water; it even improved cyclic fatigue resistance when added to distilled water at 60 °C. These results probably indicate that heat-treated systems of the new generations perform similarly well or even better at higher temperatures.

Another limitation is the focus only on the NCF of the instruments. However, a comprehensive analysis that includes the number of spirals, helical angle, blade symmetry, tip geometry, surface finishing, nickel–titanium ratio, phase transformation temperatures, and mechanical performance of the instruments is recommended for a better understanding of the behavior of each instrument system.

## 5. Conclusions

In Conclusion, Reciproc Blue and Excalibur displayed superior resistance to cyclic fatigue compared to conventional systems, indicating that reciprocating movements combined with advanced alloys enhance durability. Systems like TruNatomy and Vortex Blue also showed strong performance, with higher NCF values compared to older systems, further supporting the role of heat treatment in improving instrument flexibility and resistance.

## Figures and Tables

**Table 1:** Number of cycles until fracture (NCF) and time to fracture, expressed as mean (median; range), for second- and third-generation instruments.

	Protaper Universal	Protaper Next	2Shape	K3XF
NCF	497.0 (497.0; 393–665)	569.2 (575.0; 474–690)	576.6 (575.0; 435–750)	532.3 (525.0; 413–720)
Time	97.8 (99.5; 79–133)	113.9 (115.0; 95–138)	115.4 (115.0; 87–150)	106.46 (105.0; 83–118)

**Table 2:** Number of cycles until fracture (NCF) and time to fracture, expressed as mean (median; range), for fourth-, fifth- and sixth-generation instruments.

	BlueShaper	SlimShaper	Protaper Gold	Protaper Ultimate	Hyflex EDM	Hyflex CM	TRUshape	EDGE Endo	Vortex Blue	Trunatomy	Rotate
NCF	1050.4 (1062.5; 792–1400)	1205.7 (1206.5; 999–1396)	739.1 (762.5; 470–1020)	628.3 (613.0; 540–780)	1319.6 (1354.0; 858–2133)	1090.8 (1037.5; 658–1642)	768.6 (771.5; 502–1080)	1206.5 (1240.0; 817–1767)	1442.1 (1420.5; 1029–2000)	1567 (1440.4;1125–2245)	799.9 (775.7; 555–925)
Time	144.7 (145.0; 120–167)	126.0 (127.5; 95–168)	147.9 (152.5; 94–204)	94.2 (92.0; 81–117)	158.5 (162.5; 103–256)	131.0 (124.5; 79–197)	155.1 (154.5; 116–216)	180.5 (186.0; 123–265)	173.1 (170.5; 124–240)	188.1 (183.1; 113–285)	199.98 (193.1; 111–295)

**Table 3:** Reciprocating instrumentation systems: number of cycles until fracture (NCF) and time to fracture, expressed as mean (median; range).

	Reciproc Convén	Reciproc Blue	WaveOne Conven	WaveOne Gold	Excalibur
NCF	844.5 (837.5; 505–1075)	2454.0 (2516.5; 1967–2673)	665.9 (640.5; 531–913)	920.9 (875.5; 735–1243)	3205.3 (3096.5; 2493–4100)
Time	168.9 (167.5; 101–215)	368.1 (377.5; 295–401)	114.2 (110.0; 91–157)	157.9 (150.0; 126–213)	480.8 (464.5; 374–615)

## Data Availability

The raw data supporting the conclusions of this article will be made available by the authors on request.

## Supplementary Materials

The following supporting information can be downloaded at: https://www.mdpi.com/article/10.3390/ma17194817/s1. Supplementary Table S1.
