# Supplementary material for: Comparative Analysis of NiTi Instruments with Different Alloy Treatments
Source: Materials (Basel). 2024 Sep 30;17(19):4817. doi: 10.3390/ma17194817 (PMC11477650; doi:10.3390/ma17194817)
Supplement: Supplementary file 1 [file materials-17-04817-s001.zip › materials-3174312-supplementary.pdf]

Brand	Alloy	RPM	NCM	Size/Taper	Transverse Section
**Blue Shaper**	Pink/Gold/Blue	500	4.0	25.02%–25.06%	Convex triangular
**Slim Shaper**	Gold/Pink/Blue	500	4.0	25.04%	Triangular
**Protaper Universal**	Conventional NiTi	250	1–abr	25.08%	Convex triangular
**Protaper Next**	M-WIRE	300	2–5.2	25.06%	Off-centred rectangular
**Protaper Gold**	Gold	300	3.10	25.08%	Convex triangular
**Protaper Ultimate**	M-WIRE/Gold/Blue	300	4–5.2	25.08%	Specific parallelogram
**Vortex Blue**	Blue	500	75–368	25.04%–25.06%	Variable helical angle
**Hyflex EDM**	Gold-Blue	400	2.5	25 (variable taper)	Variable (triangular, trapezoidal and quadratic)
**Hyflex CM**	CM-Wire	500	2.5	25.04%	Variable (triangular, trapezoidal, quadratic)
**TRUShape**	Blue	300	3	25.06%	Concave triangular (active cutting)
**EEDGE Endo**	Fire-Wire NiTi	300–500	2.5–4.1	25.04%–25.06%	Parabolic
**2Shape**	T-Wire	250–400	2.5	25.06%	Triple helix
**Reciproc**	M-Wire	150ccw/30cw 300rpm	4	25.08%	S-shaped
**Reciproc Blue**	Blue	150ccw/30cw 300rpm	4	25.08%	S-shaped
**WaveOne**	M-Wire	170ccw/50cw 350rpm	4	25.08%	Modified convex triangular (apical cross section); convex triangular (coronal cross section)
**WaveOne Gold**	Gold	170ccw/50cw 350rpm	4	25.07% (variable taper)	Parallelogram
**Excalibur**	New Gold	150ccw/30cw 500rpm	4	25.05%	Italic ''S''
**Trunatomy**	Superflex	500	1.5	26.04%	Convex triangular centered
**Rotate**	Blue	300–400	2.0	25.04%	S-shaped
**K3XF**	R-Phase	300	1–mar	25.06%	Third radial land
